# PAMAM/polyhedral nanogold-modified probes with DNAase catalysis for the amperometric electrochemical detection of metastasis-associated lung adenocarcinoma transcript 1

**DOI:** 10.1186/s13036-019-0149-4

**Published:** 2019-03-06

**Authors:** Fei Liu, Tao Li, Liqun Zhang, Guiming Xiang, Dongneng Jiang, Dianji Tu, Linlin Liu, Yi Li, Chang Liu, Xiaoyun Pu

**Affiliations:** Department of Clinical Laboratory, the Second Affiliated Hospital of the Army Medical University, Chongqing, 400037 China

**Keywords:** Biosensor, Hemin, PNG, MALAT1, Long noncoding RNA (lncRNA)

## Abstract

**Abstract:**

Metastasis-associated lung adenocarcinoma transcript 1 (MALAT1), a long non coding RNA (lncRNA) present in serum, is an important biomarker for detecting hepatocellular carcinoma (HCC). However, there are some shortcomings in current detection methods. So developing other novel MALAT1 detection methods is necessary. Electrochemical biosensors using different types of nanomaterials with various advantages may provide a suitable method for detection. Here, a new strategy for MALAT1 detection was proposed based on polyhedral nanogold-polyamide-amine dendrimers (PNG-PAMAMs). The SWCNH/Au composite was used as a capture probe immobilization matrix, and PNG-PAMAM was used as a trace label for the detection probe (DP). The strategy takes advantage of the ability of the surface of PNG to bind a capture probe whose sequence contains (GGG)_3_ trimer that can bind DNAzyme hemin. Moreover, PNG may carry abundant horseradish peroxidases (HRPs) to block excess nonspecific adsorption sites, with synergistic hemin catalysis. The results show that the biosensor provides ultrasensitive detection of MALAT1 with a remarkable catalytic effect. The enhanced biosensor has a detection limit of 0.22 fmol·mL^− 1^ for MALAT1, and the linear calibration of the biosensor ranged from 1 fmol·mL^− 1^ to 100 pmol·mL^− 1^. In addition, the electrochemical biosensor has desirable qualities compared to other detectors; for instance, it is inexpensive, highly stable, and sensitive and has good reproducibility. This assay was also successfully applied to the detection of MALAT1 in serum samples, demonstrating that the technology has potential application in the detection of MALAT1 for clinical HCC diagnosis.

**Graphical Abstract:**

The schematic presentation ilustrates MALATI detection by biosensor with differential pulse stripping voltammetry. Polyhedral nanogold-PAMAM/horseradish peroxidases (PNG-PAMAM/HRP) detection probe with DNAzyme (hemin) sites was applied to determine MALATI. Signal was amplified by hemin/HRP/H_2_O_2_ catalytic system.
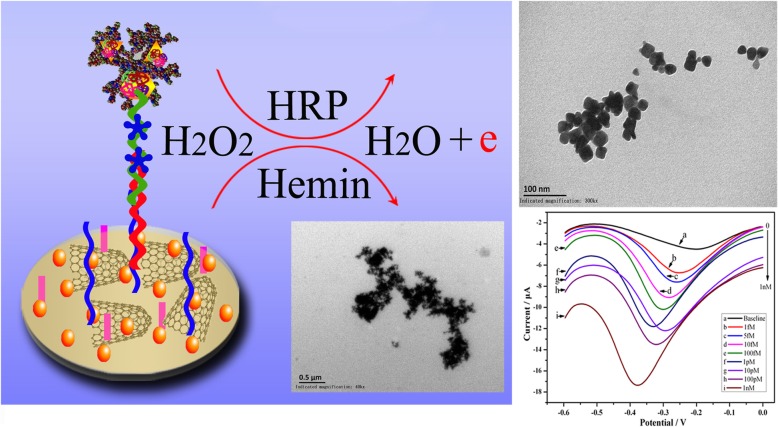

**Electronic supplementary material:**

The online version of this article (10.1186/s13036-019-0149-4) contains supplementary material, which is available to authorized users.

## Introduction

Hepatocellular carcinoma (HCC) is one of the most common types of malignant tumors worldwide, particularly in East Asian countries [1]. Metastasis-associated lung adenocarcinoma transcript 1 (MALAT1) is a long noncoding RNA (lncRNA) that is over-expressed in the serum of HCC patients. MALAT1 is a useful diagnostic biomarker for HCC and an important biomarker for HCC recurrence prediction following liver transplantation [2]. Thus, the accurate and timely detection of lncRNA, including MALAT1, is very important. Current lncRNA detection is typically based on hybridization [3]. Traditional molecular biology techniques show different advantages in the detection of lncRNA. qRT-PCR has high sensitivity and speed. Northern blot detection has strong specificity. However, PCR is only relatively quantitative and difficult to quantify absolutely. Moreover, PCR results are susceptible to interference, have a certain optimal linear range of detection, and require frequent and accurate temperature changes. Northern blot probes are radioactive and can harm the human body and pollute the environment [4]. Hence, a new accurate, sensitive and quantitative method for detecting MALAT1 is necessary.

Electrochemical nucleic acid sensors based on various nanomaterials exhibit high sensitivity and rapid performance. An electrochemical RNA biosensor that uses novel signal amplification and exhibits advantageous characteristics may provide a solution for MALAT1 detection. However, to date, there are no reports on MALAT1 detection using biosensors.

Many nanomaterials have been used to enhance the sensitivity of biosensors for noncoding RNA, such as noble metal nanomaterials [5, 6], ceriumdioxide-Au@glucose oxidase (CeO_2_-Au@GOx) [7], gold nano-particles and p-sulfonatedcalix[6]-functionalized reduced graphene oxide (Au@SCX6-rGO) [8], gold nanoparticle-Fe_3_O_4_ nanocomposite and p-sulfonated calix[8]arene-functionalized reduced graphene oxide (Fe_3_O_4_/Au @SCX8-RGO) [9], metal-organic frameworks (MOFs) [10, 11], black phosphorus nanosheets [12], and DNAzymes [13]. Single-walled carbon nanohorns (SWCNHs) and polyamide-amine (PAMAM) dendrimers are also representative materials. SWCNHs not only have the advantages of conventional carbon nanomaterials but also possess excellent catalytic properties, high purity and low toxicity and thus can be explored as a replacement for nanotubes for use in electrochemical sensing or biosensing [14], as electrochemical capacitors [15] and in H_2_O_2_ sensing [16]. PAMAM(G4.0) has a diamine core and amido-amine branching structure with hundreds of amino residues that allow these polymers to be functionalized with various noble metal nanoparticles, nucleic acids and other components [17]. Their highly branched architectures offer unique interfacial and functional advantages [18]. PAMAM has previously been used to construct biosensors to detect glucose, ethanol, and alpha fetoprotein (AFP) [19, 20].

Enzyme labels are commonly used to obtain signal amplification in sensors and biosensors on the basis of reactions with enzymes such as HRP and DNAzymes [21, 22]. DNAzymes are specific nucleic acids that are easily obtained and have a wide temperature range, high catalytic activity and recognition ability. The HRP-DNAzyme is formed by coordination between iron ions and hemin for biomolecule detection [23].

We therefore designed an electrochemical nucleic biosensor for lncRNA MALAT1 detection using polyhedral nanogold (PNG)-PAMAM/HRP as a detection probe (DP) label with a sandwich-like amplification strategy. A composite of SWCNHs was drop-cast onto electrodes and electrodeposited onto a nano-Au film. Subsequently, oligonucleotide capture probes (CP) were grafted onto the film, and complementary lncRNA was added. Following hybridization, DP labeled with PNG-PAMAM-HRP was directed to lncRNA targets, and DP has several (GGG)_3_ trimers that can serve as DNAzyme (hemin) binding sites. Hemin was used as an electronic medium to generate the current signal. Signal amplification was implemented by using a well-known method for the hemin/H_2_O_2_/HRP catalytic system. In this case, the catalytic reduction current was related to the immobilized HRP on the surface, which itself was related to the lncRNA DP surface density on the electrode. This approach has never previously been reported for lncRNA detection. Moreover, this method does not require time-consuming sample pretreatment or toxic procedures, which makes it readily adaptable to applications in HCC diagnosis and prognosis.

## Experimental

### Reagents

Gold chloride tetrahydrate (HAuCl_4_), AgNO_3_, poly-(vinylpyrrolidone) (PVP), 1,5-pentanediol, cyclohexanethiol (HT) and PAMMA (G4.0) dendrimers, poly(dimethyl diallyl ammonium chloride)(PDDA) and HRP were all purchased from Sigma Chemical Co. (St. Louis, MO, USA) (https://www.sigmaaldrich.com/china-mainland.html). Phosphate buffer (pH 5.0~8.0) was prepared using 0.1 M Na_2_HPO_4_ and 0.1 M KH_2_PO_4_. The prepared solutions were maintained at 4 °C before use. The buffer for the preparation of the lncRNA probes and target solutions was Tris-EDTA buffer (TE buffer) (10 mM Tris-Cl, pH 7.4, containing 1 mM EDTA).

The probe sequence was searched using BLAST on the NCBI website; the coincidence rate with MALAT1 was 100%, and that with other RNAs was less than 40%, indicating that the sequence is highly specific. The sulfhydryl-modified CP of MALAT1 was designed to bind the target MALAT1 near the 5′-end of the sequence. The target segments, sulfhydryl-modified CP and DP of MALAT1 were synthesized by Shanghai Sangon Biotechnology Co. (Shanghai, China) (https://www.sangon.com/services _dnasynthesis.html). miRNA-16, miRNA-21, *β-actin*,AFP, albumin (ALB) and total protein (TP) were used as interfering substances. All nucleotide sequences are shown in the supporting material (Additional file 1: Table. S1). All nucleotide segments were synthesized by Shanghai Sangon Biotechnology Co. (Shanghai, China) (https://www.sangon.com/services_dnasynthesis.html). AFP, ALB and TP were purchased from the Ningbo Ruiyuan Biological Technology Co., Ltd. (Ningbo, China) (http://www.reebio.com/products/).

Six cases of human blood serum specimens were collected from the Department of the Clinical Laboratory of Xinqiao Hospital. Informed consent was obtained from all patients.

### Apparatus

Cyclic voltammetry (CV) and differential pulse stripping voltammetry (DPV) measurements were obtained by a CHI 660d electrochemistry workstation (Shanghai CH Instruments, Shanghai, China) (http://www.chinstr.com/cpzs). The three-compartment electrochemical cell is made of a platinum wire auxiliary electrode, a saturated calomel reference electrode (SCE) and a working electrode. A glassy carbon electrode (GCE) was used as the working electrode (diameter 4 mm). Transmission electron microscopy (TEM) was carried out using a TECNAI 10 (Philips Fei Co., Hillsboro, OR). The pH was detected using a pH meter (MP 230, Mettler-Toledo, Switzerland) (https://www.mt.com/cn/zh/home.html).

### Preparation of the PDDA-SWCNHs

First, 2 mg of SWCNH powder was weighed and added to 2 mL of ddH_2_O containing 0.5% (wt) PDDA. The samples were then ultrasonicated for 16 h until completely dissolved. Subsequently, the suspension was centrifuged at 6000 rpm for 10 min to remove excess PDDA. The supernatant was discarded and the sediment collected. The sediment was resuspended in 2 mL of water and recentrifuged at 6000 rpm for 5 min, and this washing step was repeated twice. Finally, the sediment was resuspended in 2 mL of water, resulting in a stable PDDA-SWCNH solution (1 mg·mL^− 1^).

### Synthesis of polyhedral nanogold (PNG)

Polyhedral nanogold was synthesized according to the method described in the literatures [24, 25]. Briefly, 0.15 mL of AgNO_3_ solution in 1,5-pentanediol was dripped into 5.0 mL of boiling 1,5-pentanediol. Then, 3.0 mL of 0.15 M PVP and 3.0 mL of 0.05 M HAuCl_4_/1,5-pentanediol were added alternately every 30 s for 7~10 min. The mixture solution was refluxed for 1 h. After solution cooling, the particles were separated from large aggregates by centrifugation at 500 rpm for 5 min. The precipitate was washed with ethanol using a repetitive dispersion/precipitation cycle to remove excess PVP. The product was finally dispersed in 30 mL of ethanol with sonication.

### Preparation of PNG/PAMAM/HRP-DP bioconjugate

**Fig. 1:**
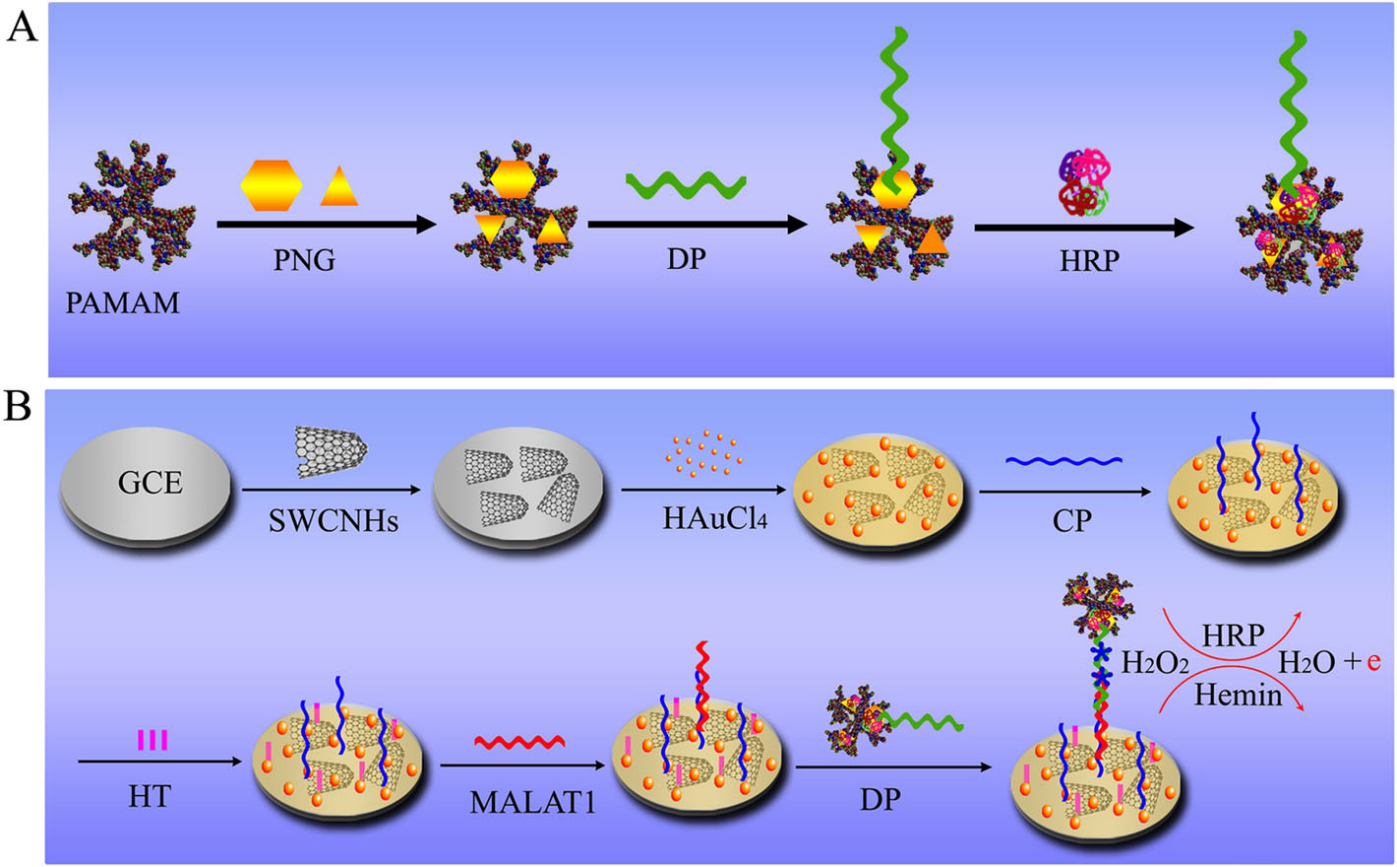
Fabrication scheme for lncRNA MALAT1 biosensor. *PAMAM* polyamide-amine dendrimer, *PNG* polyhedral nanogold, *DP* detection probe, *HRP* horseradish peroxidase, *HT* hexanethiol, *GCE* glass carbon electrode, *CP* capture probe, *MALAT1*: metastasis-associated lung adenocarcinoma transcript 1

A 1 mL aliquot of hydrated PNG was mixed with 200 μL of PAMAM (G4.0) solution overnight to form a PNG/PAMAM complex. To this solution, 50 μL of DP solution (1 μmol·mL^− 1^) was added, and the sediment was collected by centrifugation at 5000 rpm for 10 min to remove excess probe. Subsequently, 500 μL of HRP (1 mg·mL^− 1^) solution was added to block the nonspecific adsorption sites of PNG. The sediment was collected by centrifugation at 4500 rpm for 10 min to remove excess HRP (Fig. 1A). The sediment was then resuspended in 500 μL of water to prepare the DP solution, which was stored at 4 °C.

### Fabrication of biosensors

The fabrication process for the biosensor is illustrated in Fig. 1B. Before electrode modification, each bare GCE was polished with 0.3 μm and 0.05 μm alumina slurries, sonicated in deionized water, and dried with a high-purity nitrogen stream to obtain a mirror surface. Then, 8 μL of SWCNH solution was dropped onto the GCE for 8 h at 25 °C. After the electrode dried, Au particles were directly deposited in a 1% HAuCl_4_ solution. The deposition method was conducted by CV with stripping analysis. The mixed SWCNH/Au membrane was chosen as a binding matrix. MALAT1 CP was immobilized on the mixed SWCNH/Au membrane by Au-S bonds. Then, 15 μL of 2 mM HT solution was dropped onto the CP-bound electrode and incubated for 50 min to block the excess non-modified Au surface and thus weaken nonspecific absorption. The electrode was dipped in 50 mL of deionized water 3 times to wash away excess HT.

Subsequently, 15 μL of solutions of MALAT1 synthetic fragments at various concentrations was dripped onto the electrode. The reaction time was 3 h at 25 °C. Finally, 20 μL of the PNG/PAMAM/HRP-DP solution of lncRNA MALAT1 was dripped on the electrode. The incubation time was 3 h. After washing the electrode clean with ddH_2_O, the biosensor was detected in 0.1 M phosphate buffer (pH 7.4).

The basic principle of the strategy is as follows: First, PNG-PAMAM-HRP was modified with the DP of MALAT1. Target segments bound to the CP of MALAT1, which was previously used to modify the electrode. Then, PNG-PAMAM-HRP-DP bound the target segments. The more targets exist, the more probes are combined. Because DPs have many DNAzyme (hemin) binding sites, the current signal can be amplified by a hemin/H_2_O_2_/HRP catalytic system. The DPV current was related to the surface density of the DP of MALAT1 on the electrode, which enabled the quantitative detection of MALAT1.

### Experimental measurements

Electrochemical experiments were carried out in a conventional three-electrode structure for the solution system. CV characteristics for the biosensor fabrication were tested in a 5 mM [Fe(CN)_6_]^3−/4-^ solution containing 0.1 M KCl. DPVs were performed in 0.1 M phosphate buffer at 2 mL volume from - 0.6 to 0 V at a sweep rate of 50 mV/s.

## Results and discussion

### TEM analysis of nanomaterials

**Fig. 2:**
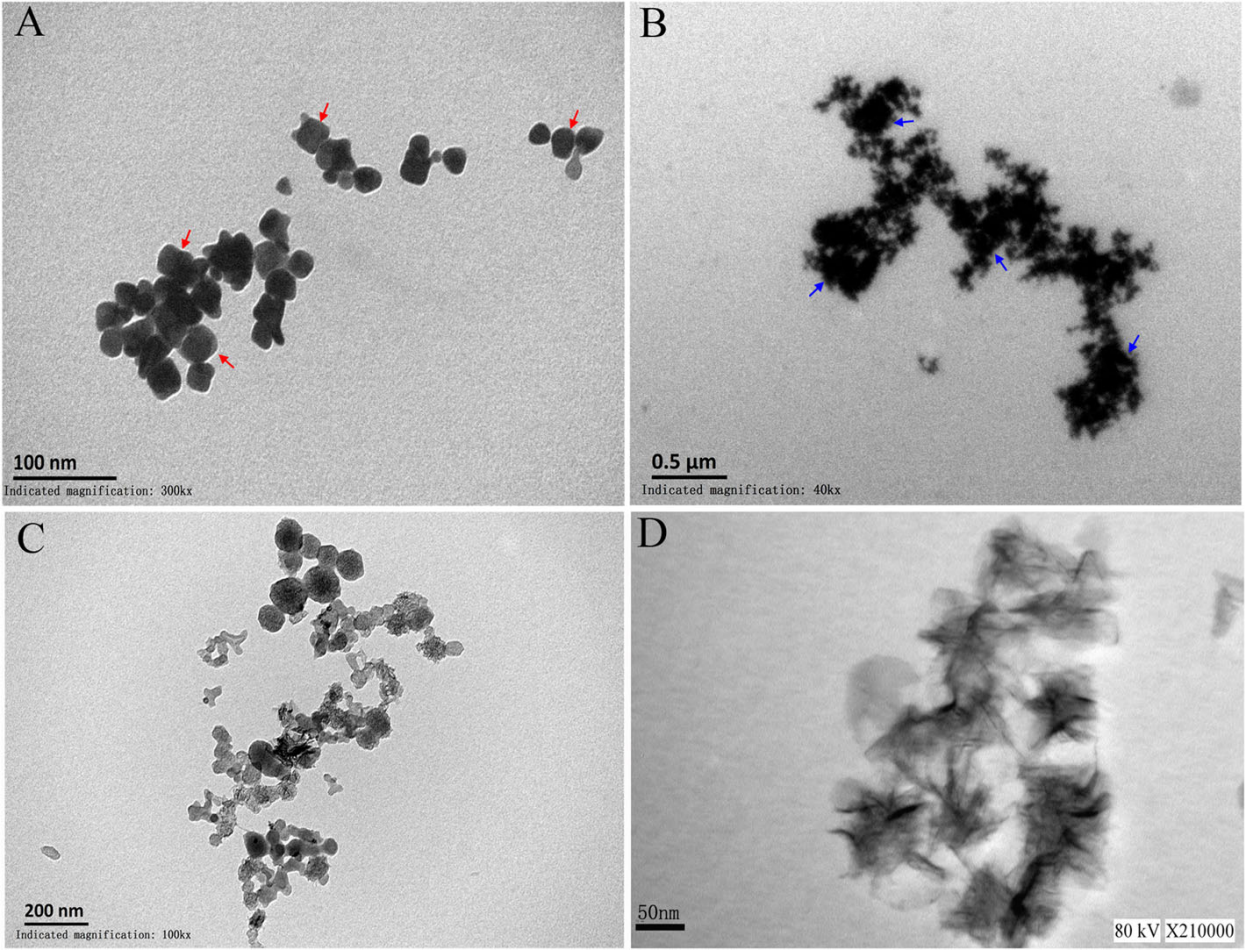
Typical TEM results for (**A**) PNG, (**B**) PAMAM-PNG, (**C**, **D**) SWCNHs (**C**: 10000×, **D**: 210000 × .). Red arrow: the typical shape of PNG, blue arrow: the PNG of binding with PAMAM

TEM images (Fig. 2A, B) reveal a typical morphology for the PNG nanomaterial. PNG nanoparticles have a controllable size or can vary in number (Fig. 2A). As indicated by the arrows in Fig. 2A, typical PNG is a hexagonal typical quadrilateral under high magnification TEM, with each field of view showing approximately 80%. Three high-power field images were collected, and 50 nanoparticles per field of view were counted to analyze the diameter of PNG [26, 27]. The density of PNG is more uniform, with a diameter range from 30 to 60 nm, confirming previous reports [28, 29].

Since PAMAM itself is an organic molecule, its conductivity is poor, and it is therefore difficult to visualize by conventional TEM. However, in conjunction with PNG, its structure is visible. PAMAM-PNG complexes have typical tree-like branches in the TEM field of view and are connected to each other. Several molecules show a reunion phenomenon. PNG nanoparticles are bound to these tree-like branches (Fig. 2B).

SWCNH is a sensor substrate modifier that was used in this work for electron microscopic characterization. As shown in Fig. 2. C, D, SWCNH hydrated by PDDA alone exhibits an aggregate surface. The SWCNHs formed dahlia-like assemblies with a diameter of approximately 100 nm. The individual SWCNH structural unit is shown clearly. Each aggregate has a convergent filament structure, consistent with the observations in previous reports [30].

### UV-vis spectra of nanomaterials

**Fig. 3:**
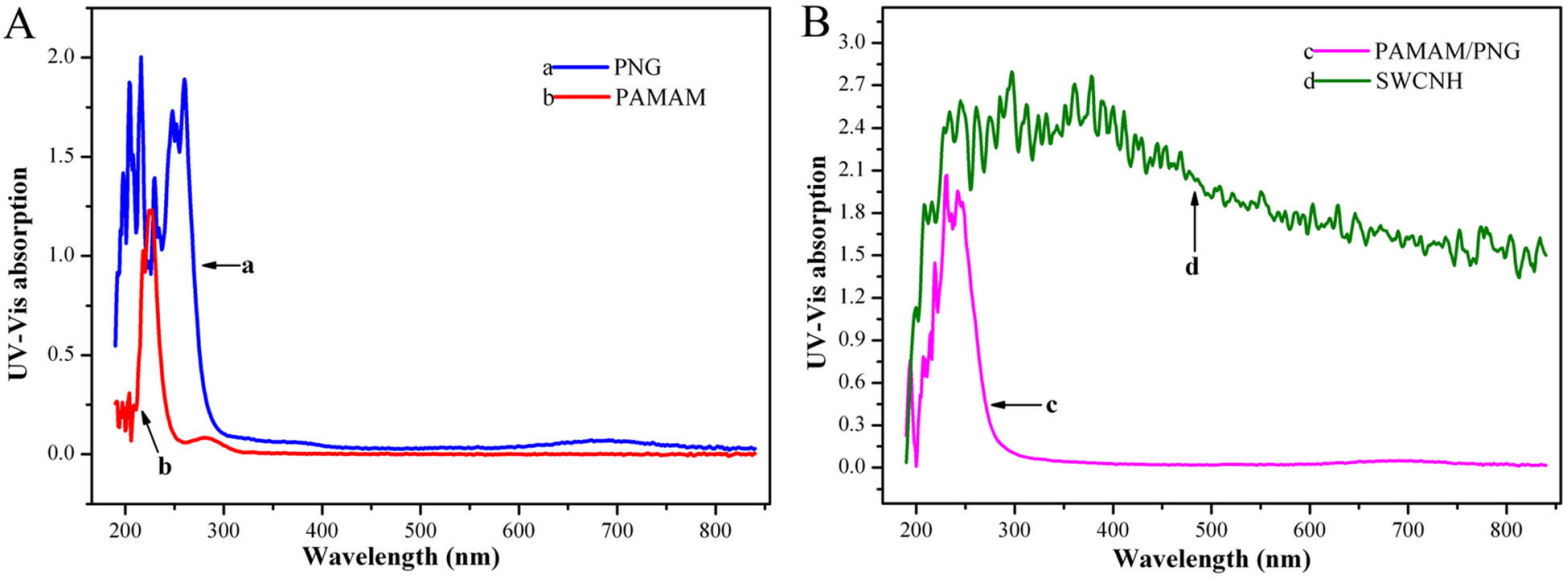
UV-Vis scans results of nanomaterials. **A**: UV-Vis of PNG (a) and PAMAM (b), **B**: UV-Vis of PAMAM-PNG complex (c) and SWCNH (d)

The UV-Vis spectra show the maximum UV absorption of these nanomaterials. The maximum UV absorption of the SWCNHs was 233–457 nm (Fig. 3A curve a). The absorption peaks of the PNG particles were 204, 216 and 248–261 nm, as corroborated by other references. PAMAM exhibited UV absorption at 226 nm (c) with a small peak at 280 nm. PAMAM-PNG exhibited peaks at 194, 215, 231, and 242 nm; the strongest signal was at 231 nm.

### CV characteristics of the MALAT1 biosensor

**Fig. 4:**
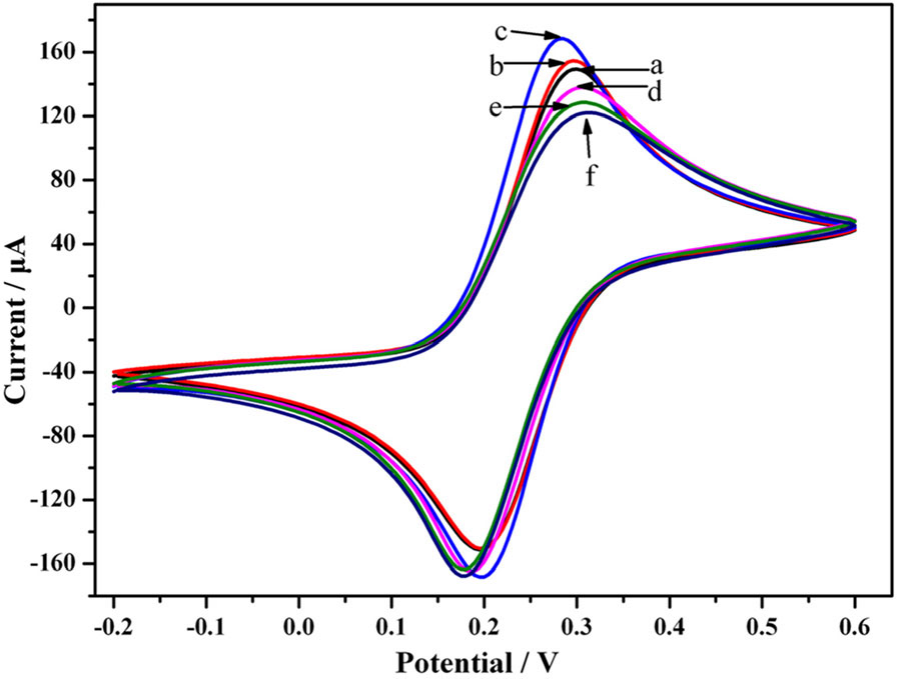
CVs of electrodes tested step by step in a 5 mM [Fe(CN)_6_]^3−/4-^ solution containing 0.1 M KCl (pH 7.4): a, bare GCE; b, deposition of SWCNHs; c, SWCNH/nano-Au; d, SWCNH/nano-Au/CP; e, SWCNH/nano-Au/CP/HT; f, SWCNH/nano-Au/CP/HT/MALAT1

CV analysis was performed in solution containing 5.0 mM K_3_[Fe(CN)_6_] /K_4_[Fe(CN)_6_]. The scan rate for CV was 50 mV/s. In Fig. 4, the bare GCE is represented by curve a, and the SWCNH nanofilms are represented by curve b, showing the reversible redox reaction. The redox peak current of the SWCNH nanofilms (curve b) increased by 3.48% (△I = 5.20 μA) compared to that of the bare GCE (curve a), suggesting that the SWCNHs exhibited low resistance. After Au electrodeposition, the current of the electrode rose 8.99% (△I = 13.9 μA, curve c), showing that Au was successfully deposited. MALAT1 CP was subsequently dropped on the electrode for 3 h at 25 °C. Compared to curve c, the current was reduced by 30.6 μA (18.16%, curve d). A considerable amount of CP bound to the Au surface on the electrode by Au-S bonding. HT was used to block excess sites. The peak currents of HT (curve e) decreased (6.82%, △I = − 9.4 μA) compared to those in curve d because of electron hindrance. The peak currents of SWCNH/Au/CP/HT/ MALAT1 (curve f) were reduced by 6.4 μA compared with those in curve e (4.98%). The CV data demonstrated that nanomaterials or nucleotide sequences (e.g., CP, MALAT1, HT) were firmly bonded to the electrode (Fig. 4). The CV characterization indicated that every step of the electrode modification was successful, implying that the lncRNA biosensor exhibited excellent loading capacity.

### Catalytic performance of the biosensor

The signal amplification of our biosensor was investigated by DPV. The results revealed that the current peak was elevated upon the addition of H_2_O_2_ due to the high catalytic activity of the G-quadruplex/hemin/HRP. The current peak clearly increased under the action of H_2_O_2_. The current response was amplified by approximately 4 times (Additional file 1: Figure S1).

The guanine bases of the TTAGGG sequence in DP first form a G-quartet. Then, four G-quartets spontaneously fold to form an intra-molecular G-quadruplex by π-π stacking, and this G-quadruplex can firmly bind to hemin [31]. The G-quadruplex/hemin structure has the catalytic activity of a hydrogen peroxide-like enzyme [32], exhibiting synergistic catalysis with HRP. Hence, the increased sensitivity is a result of several signal amplifications, revealing that the biosensor is suitable for MALAT1 detection in small sample volumes.

### Optimization of experimental parameters

The optimized pH of the reaction was determined. As shown in Additional file 1: Figure S2. A, the results indicate that the absolute value of the maximum peak current variation emerged at pH 7.4. Thus, in subsequent studies, phosphate buffer with a pH of 7.4 was considered the optimal choice. Furthermore, the concentration of H_2_O_2_ requested in phosphate buffer was checked by DPV for a series of H_2_O_2_ concentrations. The results indicated that 1.4 mM H_2_O_2_ was the best concentration (Additional file 1: Figure S2.B). Relevant data and figures are shown in the Electronic Supporting Material.

### Calibration of biosensor for MALAT1 detection

**Fig. 5:**
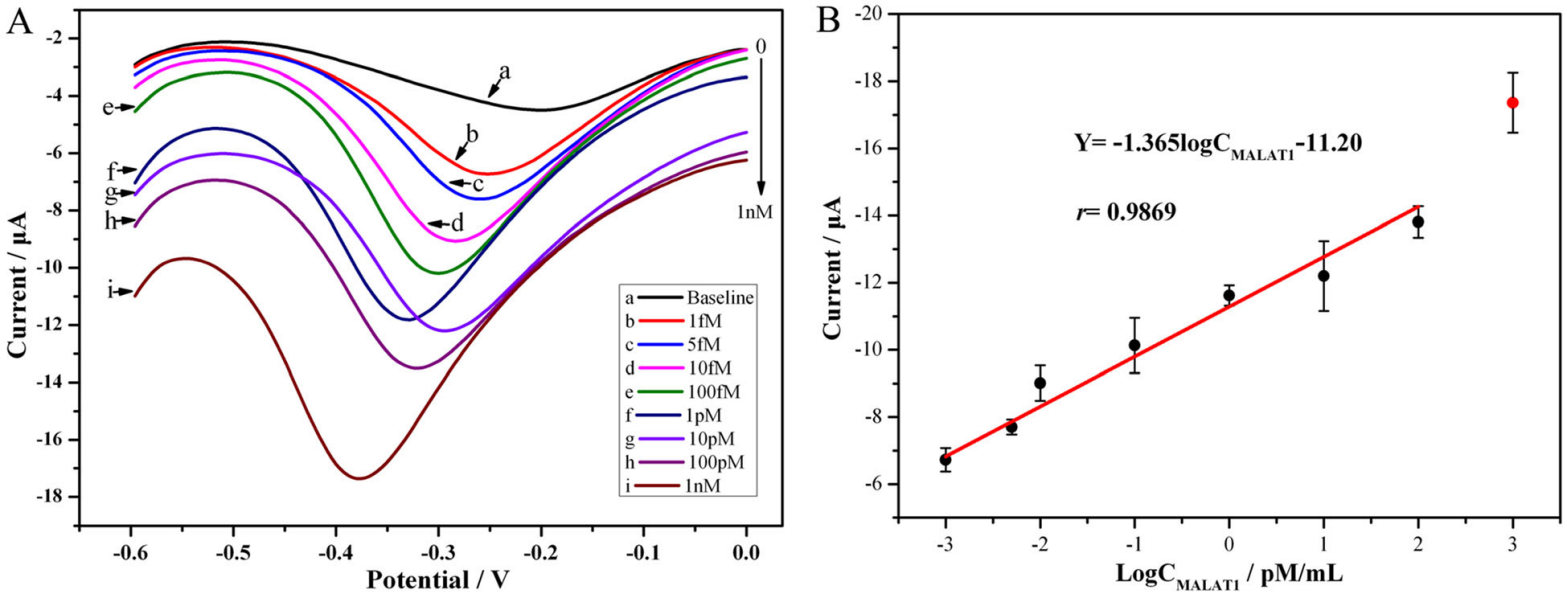
Calibration plots of the cathodic peak current response vs. the MALAT1 concentration. The inset shows DPVs for the cathodic peak at various concentrations. **A** Cathodic peak current response. a: 0, b: 1.00 × 10^− 3^, c: 5.00 × 10^− 3^, d: 1.00 × 10^− 2^, e: 1.00 × 10^− 1^, f: 1.00, g: 1.00 × 10^1^, h: 1.00 × 10^2^, i: 1.00 × 10^3^ and 1.00 × 10^4^, all units: pmol·mL^− 1^, per concentration *N* = 3. **B** Linear equation

The MALAT1 biosensor was tested using a DPV method by incubation with a series of MALAT1 concentrations. As displayed in Fig. 5, the absolute value of the cathodic peak current clearly increased as the MALAT1 concentration gradually increased. Figure 5 shows the corresponding calibration plots. The cathodic peak currents were proportional to the MALAT1 concentration from 1 fmol·mL^− 1^ to 100 pmol·mL^− 1^. However, 1 nmol·mL^− 1^ of MALAT1 is outside the linear range. A linear relationship was observed from 0 to 100 pmol·mL^− 1^. By statistical fitting, the linear equation was I = − 1.37 log CM_ALAT1_–11.20; the correlation coefficient (*r*) was 0.99, and the standard error of the concentration (*N* = 3) was 0.29. According to the formula calculation (defined as 3σ/κ, where σ is the standard deviation of the blank with a value of 0.10, and κ is the slope of linear calibration with a value of 1.37), the detection limit was 0.22 fmol·mL^− 1^.

This result indicates that MALAT1 bound to the biosensor gradually. This behavior occurs because the presence of a great excess of MALAT1 DP relative to the nanomaterials enhanced catalysis for the electron transfer and substantially promoted the current signals. The linear range is acceptable.

The table below shows the performance of other biosensors (Table. 1). Compared to related biosensors that detected other lncRNAs, for instance, MEG3, highly upregulated in liver cancer (HULC), the MALAT1 biosensor shows an excellent limit of detection (LOD) and specificity.

**Table 1:** An overview of reported nanomaterial-based electrochemical methods for the determination of lncRNAs

Method applied	Analyte detection	Materials used	LOD	References
DPV	MEG3	F_e3_O_4_@C biocomposite	0.25 fM	[33]
ECL	HULC	Au@Ag/GQDs complex	0.3 fM	[34]
DPV	HULC	PtPd BND/BNF@GO	0.25 fM	[35]
DPV	NEAT1	Au/Rh-HNP@SWCNT	0.89 fM	[6]
Fluorescent or electrochemical	SChLAP1	DNA-templated CuNBs	500 fM/100 fM	[36]
Amperometric	MALAT1	SA-Dynabeads/TMB	10 cells/mL	[37]
DPV	MALAT1	PNG/PAMAM/Hemin	0.22 fM	This work

### Specificity, reproducibility, and stability of MALAT1 biosensor

To investigate whether the collected current response was produced by probe-target specific interaction or by a nonspecific nucleotide interaction, a specificity experiment was performed. The MALAT1 biosensor was reacted with different interfering substances: miRNA16, miRNA21, *actin*, AFP, ALB, TP, MALAT1 and MALAT1 mixture. ALB and TP are the most abundant proteins in human serum. To better approximate the real blood environment, albumin has been used as an interfering substance to test specificity [38]. Because the nonspecific binding sites of the biosensor are blocked by HT and washing is performed at each step, additional interfering substances cannot absorb on the electrode. Its nonspecific adsorption exhibits restrictive saturation. The concentration of each interfering substance was set to 50 times that of the target.

**Fig. 6:**
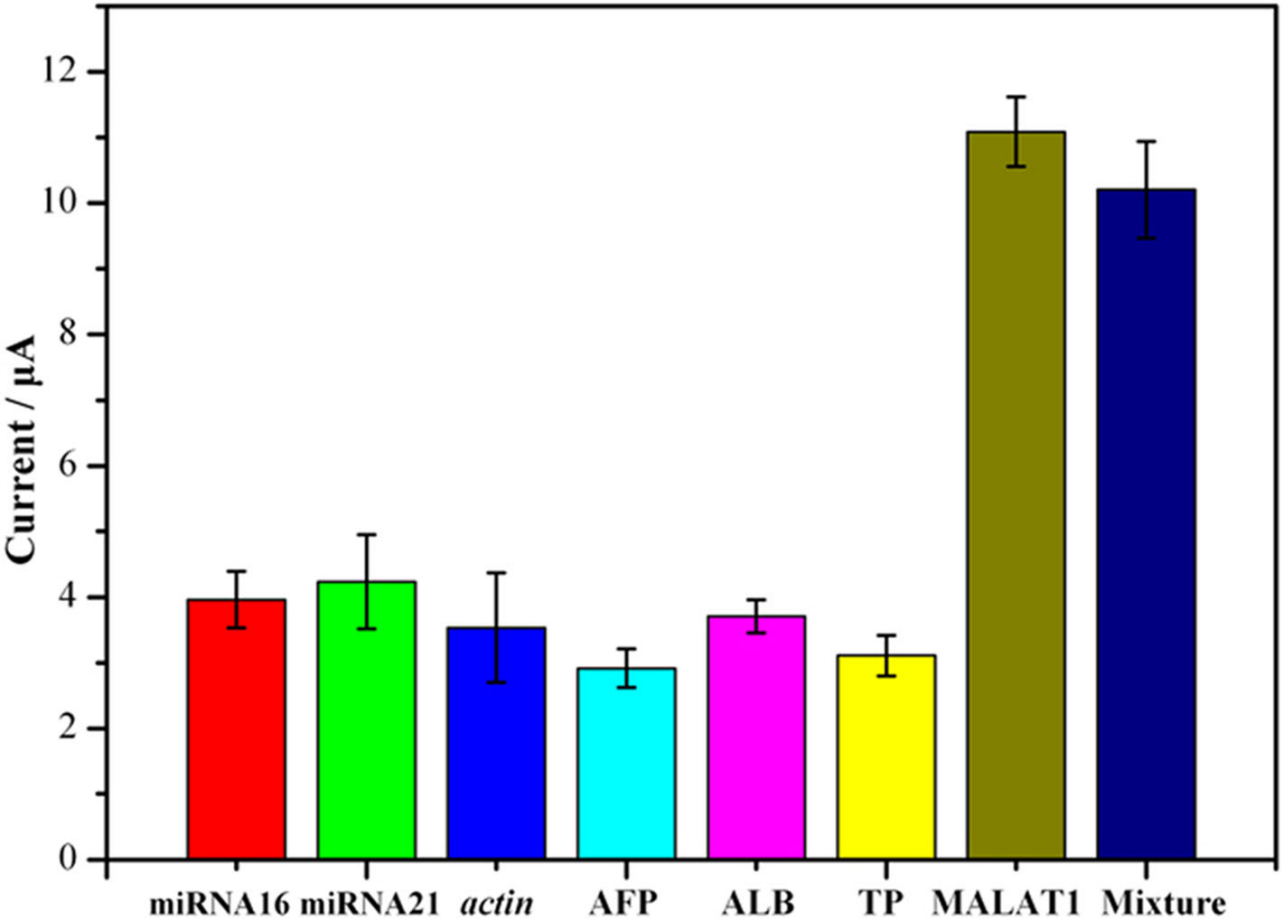
Specificity of MALAT1 biosensor with 50 pmol of miRNA16, 50 pmol of miRNA21, 50 pmol of *actin*, 5 ng of AFP, 10 ng of ALB (albumin), 10 ng of TP (total protein), 1 pmol of MALAT1 and a mixture (1 pmol MALAT1, 50 pmol of miRNA21, 50 pmol of miRNA16, 50 pmol of *actin*, 5 ng of AFP, 10 ng of ALB and TP, per interfering substance *N* = 3)

The presence of RNA (50 pmol of miRNA16, miRNA21 and *actin*) and protein (5 ng AFP, 10 ng ALB,10 ng of GLO) caused minimal current changes even at a 50-fold excess relative to MALAT1, similar to that of a blank sample. However, incubation with perfectly matched target lncRNA MALAT1 or its mixture at an even lower concentration (50-fold, 1 pmol) resulted in an obvious elevation in the current response (Fig. 6). The disparity is statistically significant, as calculated using SPSS 19.0 (*P* < 0.05). The results show that the MALAT1 biosensor has excellent specificity.

The results revealed MALAT1 detection with acceptable reproducibility. Moreover, stability tests of the MALAT1 biosensor also showed acceptable results (see Electronic Supporting Material).

### Synthetic serum sample analysis

Recovery tests were carried out to assess the feasibility of the biosensor for use [39, 40]. First, several serum samples were prepared. Different concentrations of MALAT1 synthetic segments were added to the serum to form a concentration gradient: 0.5, 1, 5, 10, 20, and 40 pmol·mL^− 1^. Then, they were tested using the biosensor. The data in Table 2 show recoveries between 99.2 and 110.3% (Table. 2). The results of the synthetic segment mixed serum sample test obtained were acceptable. Thus, the strategy is feasible and meets the requirements for practical detection.

**Table. 2:** Recovery analysis for MALAT1 in serum samples

Serum sample	Added (pmol)	Response(μA)	Found (pmol)	Recovery (%)
1	0.5	−10.82	0.527	105.4
2	1	−11.26	1.103	110.3
3	5	−12.16	5.054	101.1
4	10	−12.56	9.915	99.2
5	20	−12.99	20.593	103.0
6	40	−13.39	40.271	100.7

### Detection of lncRNA MALAT1 in HCC cell lines

The proposed RNA biosensor was investigated by standard addition methods in HCC cancer cell lines. HepG2 and Hep3B are classical and common liver cancer cell lines. Therefore, both cell lines were employed in the real application test. First, we used *rt*-PCR to provide a quantitative comparison (Additional file 1: Figure S3). The MALAT1 concentration of HepG2 and Hep3B were subsequently detected by the proposed biosensor. The results revealed that the trends in the biosensor results are very similar to those detected by *rt*-PCR (Additional file 1: Table. S2), which indicated that the proposed biosensor was feasible for the determination of lncRNA MALAT1 and could satisfy the need for practical analyses.

Although the sensor exhibits good detection characteristics, the narrow linear range is its own defect (less than 1 nmol·mL^− 1^). This characteristic requires follow-up experiments for improvement.

## Conclusions

In conclusion, we have demonstrated the applicability of a sensitive amperometric biosensor for the detection of the biomarker MALAT1 based on nano-SWCNHs and a nano-Au composite as CP immobilization matrices and PNG-PAMAM-HRP as a trace label. This PNG-PAMAM and HRP nanomaterials can provide a favorable microenvironment for biomolecules and effectively maintain their activities. Moreover, the large surface area, rich active sites, and good electrical properties make PNG-PAMAM a promising nanomaterial for signal-amplifying applications. Due to the high loading of CP and the double catalytic effect of DP, the sensitivity of the nucleic biosensor for MALAT1 was enhanced with a detection limit of 0.22 fmol·mL^− 1^, which is below the commonly accepted concentration threshold in clinical diagnosis. The linear calibration of the biosensor ranged from 1 fmol·mL^− 1^ to 100 pmol·mL^− 1^. This electrochemical biosensor shows advantages such as low cost, high stability, high sensitivity and good reproducibility. This assay was also successfully applied to the detection of MALAT1 in serum samples, indicating that the biosensor has potential for application in the detection of the lncRNA MALAT1 for clinical HCC diagnosis.

## Additional file

Additional file 1: **Table S1.** The sequence of MALAT1 probes, target, primers and nucleotide acids interfere substance. **Figure S1.** Catalytic activity in 2 mL of phosphate buffer (pH 7.0): a, without H_2_O_2_; b, with H_2_O_2_ (1 mM). **Figure S2.** Optimization of (A) pH of MALAT1CP reaction (*N* = 3) and (B) concentration of H_2_O_2_ between 0 and 1.8 mM·L^− 1^ (*N* = 3). **Figure S3** Expression of lncRNA MALAT1 in HCC cell lines (*N* = 3). **Table S2** Determination of MALAT1 concentration of HCC cell lines with proposed biosensors. (DOCX 218 kb)

## Abbreviations

ALB: Albumin
CP: Capture probes
CV: Cyclic voltammetric
DP: Detection probe
DPV: Differential pulse stripping voltammetry
GCE: Glassy carbon electrode
HCC: Hepatocellular carcinoma
HRPs: Horseradish peroxidases
HT: Cyclohexanethiol
lncRNA: long non-coding RNA
LOD: Limit of detection
MALAT1: Metastasis-associated lung adenocarcinoma transcript 1
MOFs: metal-organic frameworks
PNG-PAMAM: polyhedral nanogold-polyamide amine dendrimer
PVP: Poly-(vinylpyrrolidone)
SCE: Saturated calomel reference electrode
SWCNHs: Single-walled carbon nanohorns
TEM: Transmission electron microscopy
TP: Total protein

## References

[1] Siegel RL, Miller KD, Jemal A (2017). Cancer statistics, 2017. CA Cancer J Clin.

[2] Konishi H, Ichikawa D, Yamamoto Y, Arita T, Shoda K, Hiramoto H, Hamada J, Itoh H, Fujita Y, Komatsu S, Shiozaki A, Ikoma H, Ochiai T, Otsuji E (2016). Plasma level of metastasis-associated lung adenocarcinoma transcript 1 is associated with liver damage and predicts development of hepatocellular carcinoma. Cancer Sci.

[3] Cissell KA, Deo SK (2009). Trends in microRNA detection. Anal Bioanal Chem.

[4] Kong W, He LL, Coppola M, Guo JP, Esposito NN, Coppola D, Cheng JQ (2010). MicroRNA-155 regulates cell survival, growth, and Chemosensitivity by targeting FOXO3a in breast Cancer. J Biol Chem.

[5] Kilic T, Erdem A, Ozsoz M, Carrara S (2018). microRNA biosensors: opportunities and challenges among conventional and commercially available techniques. Biosens Bioelectron.

[6] Liu F, Xiang G, Zhang L, Jiang D, Liu L, Li Y, Liu C, Pu X (2015). A novel label free long non-coding RNA electrochemical biosensor based on green L-cysteine electrodeposition and au-Rh hollow nanospheres as tags. RSC Adv.

[7] Sun X, Wang H, Jian Y, Lan F, Zhang L, Liu H, Ge S, Yu J (2018). Ultrasensitive microfluidic paper-based electrochemical/visual biosensor based on spherical-like cerium dioxide catalyst for miR-21 detection. Biosens Bioelectron.

[8] Zhao H, Liu F, Wu S, Yang L, Zhang Y-P, Li C-P (2017). Ultrasensitive electrochemical detection of Dicer1 3′UTR for the fast analysis of alternative cleavage and polyadenylation. Nanoscale.

[9] Zhao H, Liu F, Lu Y, Jin L, Tan S, Zhang Y, Li C-P (2019). Ultrasensitive electrochemical detection of alternative cleavage and polyadenylation of CCND2 gene at the single-cell level. Sensors Actuators B Chem.

[10] Wang H, Jian Y, Kong Q, Liu H, Lan F, Liang L, Ge S, Yu J (2018). Ultrasensitive electrochemical paper-based biosensor for microRNA via strand displacement reaction and metal-organic frameworks. Sens Actuators B Chem.

[11] Wang H, Jian Y, Kong Q, Liu H, Lan F, Liang L, Ge S, Yu J (2018). Ultrasensitive electrochemical paper-based biosensor for microRNA via strand displacement reaction and metal-organic frameworks. Sensors Actuators B Chem.

[12] Zhou J, Li Z, Ying M, Liu M, Wang X, Wang X, Cao L, Zhang H, Xu G. Black phosphorus nanosheets for rapid microRNA detection. Nanoscale. 2018. 10.1039/c7nr08900g. 10.1039/c7nr08900g 29488527

[13] Zhang H, Wang K, Bu S, Li Z, Ju C, Wan J (2018). Colorimetric detection of microRNA based on DNAzyme and nuclease-assisted catalytic hairpin assembly signal amplification. Mol Cell Probes.

[14] Yang L, Ran X, Cai L, Li Y, Zhao H, Li C-P (2016). Calix[8]arene functionalized single-walled carbon nanohorns for dual-signalling electrochemical sensing of aconitine based on competitive host-guest recognition. Biosens Bioelectron.

[15] Wang X, Lou M, Yuan X, Dong W, Dong C, Bi H, Huang F (2017). Nitrogen and oxygen dual-doped carbon nanohorn for electrochemical capacitors. Carbon.

[16] Bracamonte MV, Melchionna M, Giuliani A, Nasi L, Tavagnacco C, Prato M, Fornasiero P (2017). H2O2 sensing enhancement by mutual integration of single walled carbon nanohorns with metal oxide catalysts: the CeO2 case. Sensors Actuators B Chem.

[17] Sadekar S, Ghandehari H (2012). Transepithelial transport and toxicity of PAMAM dendrimers: implications for oral drug delivery. Adv Drug Deliver Rev.

[18] Esfand R, Tomalia DA (2001). Poly(amidoamine) (PAMAM) dendrimers: from biomimicry to drug delivery and biomedical applications. Drug Discov Today.

[19] Senel M, Nergiz C, Cevik E (2013). Novel reagentless glucose biosensor based on ferrocene cored asymmetric PAMAM dendrimers. Sensor Actuat B-Chem.

[20] Bahadır EB, Sezgintürk MK (2016). Poly(amidoamine) (PAMAM): an emerging material for electrochemical bio(sensing) applications. Talanta.

[21] Liu Y, Liu X, Guo Z, Hu Z, Xue Z, Lu X (2017). Horseradish peroxidase supported on porous graphene as a novel sensing platform for detection of hydrogen peroxide in living cells sensitively. Biosens Bioelectron.

[22] Moreno-Guzmán M, García-Carmona L, Molinero-Fernández Á, Cava F, López Gil MÁ, Escarpa A (2017). Bi-enzymatic biosensor for on-site, fast and reliable electrochemical detection of relevant D-amino acids in bacterial samples. Sensors Actuators B Chem.

[23] Alizadeh N, Salimi A, Hallaj R. Hemin/G-Quadruplex horseradish peroxidase-mimicking DNAzyme: principle and biosensing application. Adv Biochem Eng Biotechnol. 2017. 10.1007/10_2017_37. 10.1007/10_2017_37 29143069

[24] Seo D, Park JC, Song H (2006). Polyhedral gold nanocrystals with O-h symmetry: from octahedra to cubes. J Am Chem Soc.

[25] Seo D, Yoo CI, Park JC, Park SM, Ryu S, Song H (2008). Directed surface overgrowth and morphology control of polyhedral gold nanocrystals. Angew Chem Int Edit.

[26] Emam HE, Zahran MK, Ahmed HB (2017). Generation of biocompatible nanogold using H2O2–starch and their catalytic/antimicrobial activities. Eur Polym J.

[27] Ahmed HB, Abdel-Mohsen AM, Emam HE (2016). Green-assisted tool for nanogold synthesis based on alginate as a biological macromolecule. RSC Adv.

[28] Kovács G, Fodor S, Vulpoi A, Schrantz K, Dombi A, Hernádi K, Danciu V, Pap Z, Baia L (2015). Polyhedral Pt vs. spherical Pt nanoparticles on commercial titanias: is shape tailoring a guarantee of achieving high activity?. J Catal.

[29] Judy JD, Tollamadugu NVKVP, Bertsch PM (2012). Pin oak (Quercus palustris) leaf extract mediated synthesis of triangular, polyhedral and spherical gold nanoparticles. Advances in Nanoparticles.

[30] Yuge R, Nihey F, Toyama K, Yudasaka M (2017). Carbon nanotubes forming cores of fibrous aggregates of carbon nanohorns. Carbon.

[31] Huppert JL (2008). Four-stranded nucleic acids: structure, function and targeting of G-quadruplexes. Chem Soc Rev.

[32] Golub E, Albada HB, Liao WC, Biniuri Y, Willner I (2016). Nucleoapzymes: hemin/G-Quadruplex DNAzyme-aptamer binding site conjugates with superior enzyme-like catalytic functions. J Am Chem Soc.

[33] Li XY, Peng G, Cui F, Qiu QY, Chen XJ, Huang H (2018). Double determination of long noncoding RNAs from lung cancer via multi-amplified electrochemical genosensor at sub-femtomole level. Biosens Bioelectron.

[34] Li JJ, Shang L, Jia LP, Ma RN, Zhang W, Jia WL, Wang HS, Xu KH (2018). An ultrasensitive electrochemiluminescence sensor for the detection of HULC based on au@ag/GQDs as a signal indicator. J Electroanal Chem.

[35] Liu F, Xiang G, Jiang D, Zhang L, Chen X, Liu L, Luo F, Li Y, Liu C, Pu X (2015). Ultrasensitive strategy based on PtPd nanodendrite/nano-flower-like@GO signal amplification for the detection of long non-coding RNA. Biosens Bioelectron.

[36] Koo KM, Carrascosa LG, Trau M (2018). DNA-directed assembly of copper nanoblocks with inbuilt fluorescent and electrochemical properties: application in simultaneous amplification-free analysis of multiple RNA species. Nano Res.

[37] Islam MN, Moriam S, Umer M, Phan HP, Salomon C, Kline R, Nguyen NT, Shiddiky MJA (2018). Naked-eye and electrochemical detection of isothermally amplified HOTAIR long non-coding RNA. Analyst.

[38] Zhang H, Zuo F, Tan X, Xu S, Yuan R, Chen S (2018). A novel electrochemiluminescent biosensor based on resonance energy transfer between poly(9,9-di-n-octylfluorenyl-2,7-diyl) and 3,4,9,10-perylenetetracar-boxylic acid for insulin detection. Biosens Bioelectron.

[39] Zhang SL, Zhang L, Zhang X, Yang PH, Cai JY (2014). An efficient nanomaterial-based electrochemical biosensor for sensitive recognition of drug-resistant leukemia cells. Analyst.

[40] Li Y, Deng J, Fang LC, Yu KK, Huang H, Jiang LL, Liang WB, Zheng JS (2015) A novel electrochemical DNA biosensor based on HRP-mimicking hemin/G-quadruplex wrapped GOx nanocomposites as tag for detection of Escherichia coli O157:H7. Biosens Bioelectron 63:1–6. 10.1016/j.bios.2014.07.012. 10.1016/j.bios.2014.07.012 25048446

