# Supplementary material for: PAMAM/polyhedral nanogold-modified probes with DNAase catalysis for the amperometric electrochemical detection of metastasis-associated lung adenocarcinoma transcript 1
Source: J Biol Eng. 2019 Mar 6;13:21. doi: 10.1186/s13036-019-0149-4 (PMC6404345; doi:10.1186/s13036-019-0149-4)
Supplement: Supplementary file 1 — Table S1. The sequence of MALAT1 probes, target, primers and nucleotide acids interfere substance. Figure S1. Catalytic activity in 2 mL of phosphate buffer (pH 7.0): a, without H2O2; b, with H2O2 (1 mM). Figure S2. Optimization of (A) pH of MALAT1CP reaction (N = 3) and (B) concentration of H2O2 between 0 and 1.8 mM·L− 1 (N = 3). Figure S3 Expression of lncRNA MALAT1 in HCC cell lines (N = 3). Table S2 Determination of MALAT1 concentration of HCC cell lines with proposed biosensors. (DOCX 218 kb) [file 13036_2019_149_MOESM1_ESM.docx]

**PAMAM/polyhedral nanogold modified probes with DNAase catalysis for amperometric electrochemical detection of metastasis-associated lung adenocarcinoma transcript 1**

Fei Liu, Tao Li, Liqun Zhang, Guiming Xiang, Dongneng Jiang, Dianji Tu, Linlin Liu, Yi Li, Chang Liu , Xiaoyun Pu*

Department of Clinical Laboratory, the Second Affiliated Hospital of the Army Medical University, Chongqing 400037, China.

**1. Experiment**

**1.1 Reagents and Apparatus**

Total RNA extraction reagent and PrimeScript TM RT reagent Kit with gDNA Eraser were purchased from Takara Biomedical Technology Co. Ltd (DaLian, China,). 294 mM (1%) H_2_O_2_ was diluted with water from 8.82 M (30%) maternal H_2_O_2_ solution. Phosphate buffer (pH 5.0~8.0) was prepared using 0.1 M Na_2_HPO_4_ and 0.1 M KH_2_PO_4_. The sequence of MALAT1 probes, target and miRNA16, miRNA21 were shown in Table S1.

The primers of MALAT1 were designed using Primer Premier 5.0 Software. The sequences are listed in Table S1.

**Table. S1** The sequence of MALAT1 probes, target, primers and nucleotide acids interfere substance

| **Name** | **Sequence** |
| --- | --- |
| CP | 5′-AACTGTAAACCTGTG-(CH_2_)_6_-SH-3′ |
| target RNA | 5′-CACAGGTTTACAGTTTATAGAAACTAGAGCAGTTCTCACG-3′ |
| DP | 5′-SH-(CH_2_)_6_-(ttaggg)_4_cgtgagaactgctctacgtgagaactgctc  ta-3′ |
| Primer-F | 5′-TGACTCAAGGTGTAACAGAA-3′ |
| Primer-R | 5′-GGACATCTCTTCCACAGAC-3′ |
| miRNA-16 | 5′-CTCAACTGGTGTCGGGAGTCGGCAATTCAGTTGAGCGCCAATA-3′ |
| miRNA-21 | 5′-tgtcgggtagcttatcagactgatgttgac-3′ |
| *β-actin* | 5′-TGACGTGGACATCCGCAAAG-3′ |

CP: Capture Probe, DP: Detection Probe, Primer-F: Primer forward, Primer-R: Primer reversed.

Reagent addition of H_2_O_2_ was carried out by microsampler (Zhenhai Glass Co., Ltd., Ningbo, China). Pulse stripping voltammetry (DPV) measurements were performed using a CHI 660d electrochemistry workstation (Shanghai CH Instruments, Shanghai, China).

**1.2 Catalytic performance measurement**

Prepared pH 7.0 phosphate buffer (0.1M) for close to pH range of human body. Then, add 2mL phosphate buffer (0.1M) into the 5 mL test cup. Put the prepared biosensors into the test cup. Then 6.8 μLof 294 mM (1%) of H_2_O_2_ was added into 2mL phosphate buffer with 10 μL microsampler. The final conectration of H_2_O_2_ is 1.0 mM (0.003%) in phosphate buffer. The response of the biosensor was measured using DPV from -0.6 to 0 V by CHI 660d electrochemistry workstation.

**1.3 Total RNA extraction**

Total RNA was extracted according to the instructions of total RNA extraction reagent. The experimental procedure is as follows:

Collected 1×10^7^ cells of HepG2 and Hep3B cells, 1 mL of RNAiso Plus was added into cancer cells, incubated 5min at room temperature; Add 0.2 mL of chloroform and 1 mL of Trizol, at room temperature for 3 min; Centrifugation, 12000 g, 15 min. Transfer the upper aqueous phase to another new EP tube, add an equal volume of isopropanol and invert the centrifuge tube and mix well, place at room temperature for 10 min, centrifuge at 4 ℃, 12000 g, 10 min; The supernatant was carefully discarded, and the RNA pellet was washed with 1 mL of 75% ethanol (DEPC water), centrifuged at 4 ℃, 7500 g, 5 min. Discarding the ethanol, the RNA precipitate is dried at room temperature for 5-10 min; Take appropriate amount of RNase-free H_2_O to dissolve RNA, measure OD260 and OD280 by UV spectrophotometer, and calculate RNA content and purity.

**1.4 Reverse *rt*-PCR experiment**

Reverse transcription system and method are performed according to the instructions of the PrimeScriptTM RT reagent Kit with gDNA Eraser. Add the following reagents to get cDNA:

| Material | Volume (μL) |  |  |
| --- | --- | --- | --- |
| PrimeScriptRT Enzyme Mix I | 1.0 | |  |
| RT Primer Mix | 1.0 | |  |
| Total RNA | 2.0 | |  |
| 5×PrimerScript Buffer | 4.0 | |  |
| Rnase Free ddH_2_O | 12.0 | |  |
| Total volume | 20.0 | | |

42 ℃, 2 min, centrifugation,.  Mix well, centrifuge, 37 ℃, 15 min; 85 ℃, 5 s, -20 ℃ reserved.

The PCR reaction system is as follows:

| Material | Volume(μL) |
| --- | --- |
| SYBR Premix Ex Taq II | 10.0 |
| Primer F（10μM） | 1.0 |
| Primer R（10μM） | 1.0 |
| ROX Reference Dye | 0.5 |
| cDNA | 1.0 |
| Rnase Free ddH_2_O | 11.5 |
| Total volume | 25.0 |

Real-Time PCR reaction procedure: 95℃, 5min; PCR cycle (×40 cycles): 94℃, 30s; 54℃, 20s; 72℃, 30s (fluorescence signal collection); 72℃, 5min.

**2. Results**

**2.1 Catalytic performance of the biosensor**

The signal amplification of our biosensor was based on PAMAM-PNG/hemin/HRP. To analyze the catalytic effect on H_2_O_2_, H_2_O_2_ was added to phosphate buffer (2 mL, pH 7.0), and the current of the biosensor was detected by DPV. The results revealed that the current peak was elevated upon the addition of H_2_O_2_ due to the high catalytic activity of the G-quadruplex/hemin/HRP. The current peak clearly increased under the action of H_2_O_2_. The current response was amplified about 4-times (Fig. S1), demonstrating a catalytic effect that significantly surpasses previous reports [1,2]. Hence, a higher sensitivity effect is a result of several signal amplifications, revealing that the biosensor is suitable for MALAT1 detection in small sample volumes.

**
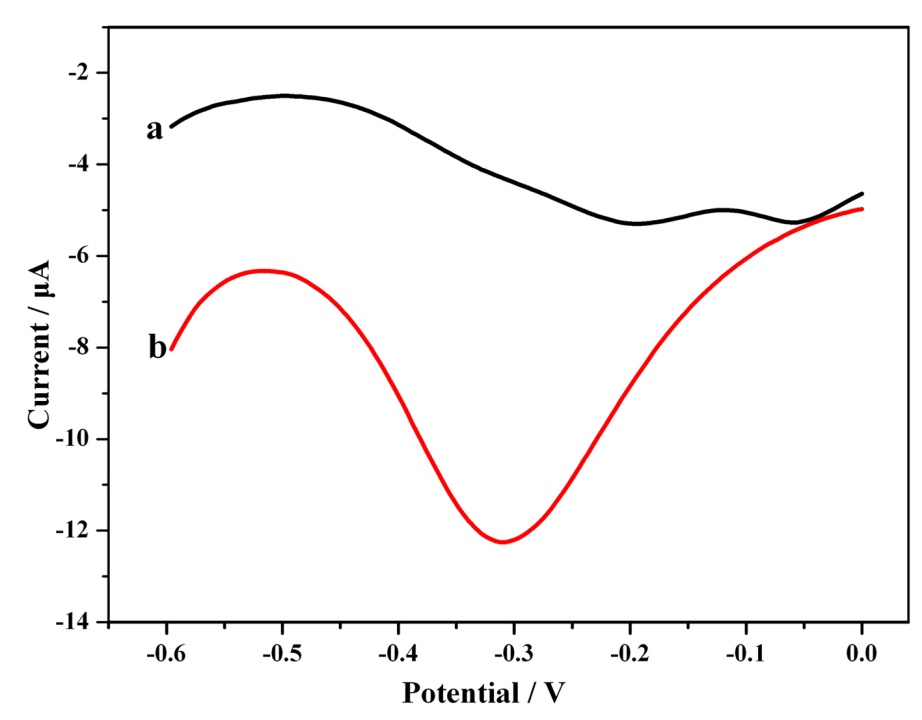
**

**Fig.S1** Catalytic activity in 2 mL of phosphate buffer (pH 7.0): a, without H_2_O_2_; b, with H_2_O_2_ (1 mM).

**2.2 Optimization of experimental parameters**

The optimized pH for reaction phosphate buffer was investigated. A complete state biosensor binding with 1 pmol⋅mL^-1^ MALAT1 was put into phosphate buffer (0.1 M). The volume is 2 mL and contains H_2_O_2_. DPV method was used to test current changes. The pH of the working buffer was ranging from 5.0 to 8.0. And the proposed biosensors corresponding to different pH were measured. Fig. S2A shows the optimization results. At pH 7.4, the biosensor gained the maximum absolute value of current response. Thus, phosphate buffer with pH 7.4 was considered the optimal condition and was applied as the working buffer in subsequent studies.

Moreover, the concentration of H_2_O_2_ in the detection solution was tested by DPV for various concentrations of H_2_O_2_. This detection was based on the biosensor reaction with 1 pmol⋅mL^-1^ MALAT1 (Fig. S2B). The results revealed that the absolute value of the current responses increased as the concentration of H_2_O_2_ increased from 0 mM. As concentration of H_2_O_2_ reaches 1.4 mM, it obviously enters platform stage. Even at 1.8 mM H_2_O_2_ the biosensor current was inhibited. Excess H_2_O_2_ may lead to this phenomenon. Therefore, 1.4 mM H_2_O_2_ was chosen as the best concentration for next experiments.


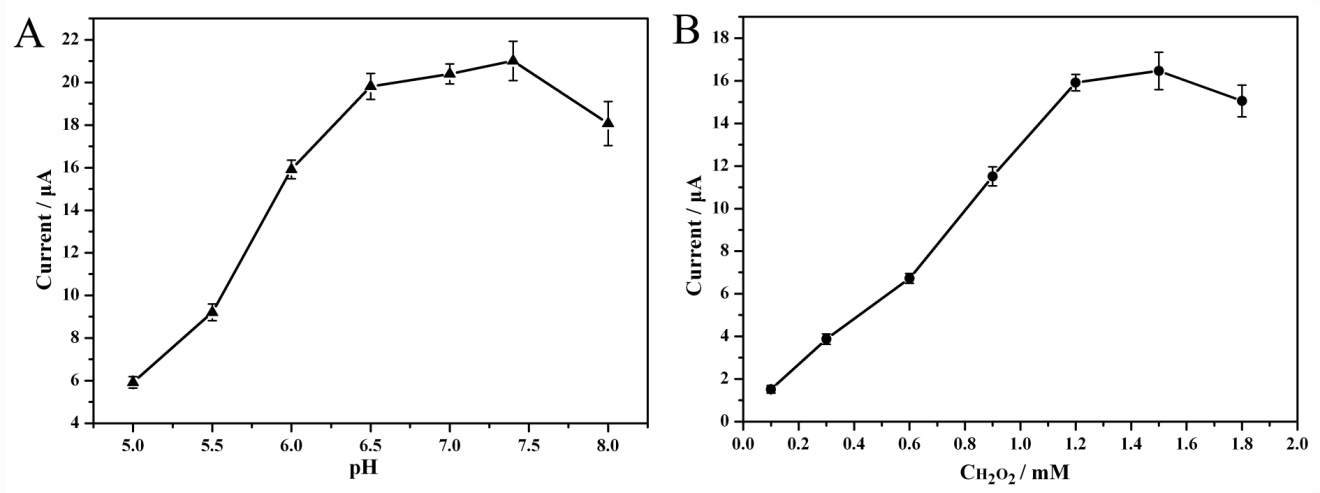


**Fig. S2** Optimization of (A) pH of MALAT1CP reaction (N=3) and (B) concentration of H_2_O_2_ between 0 and 1.8 mM⋅L^-1^ (N=3).

**2.3 Reproducibility and stability of proposed MALAT1 biosensor**

The intra-assay and inter-assay reproducibility of the proposed MALAT1 biosensor was explored. The intra-assay reproducibility was 4.27%, as analyzed from the response of 1 pmol⋅mL^-1^ MALAT1 in the same batch with five biosensors. The inter-assay reproducibility was tested at a sole MALAT1 concentration (1 pmol⋅mL^-1^) by 5 RNA biosensors at the same electrode in batches. The relative standard deviation (R.S.D.) of the inter-assay was 2.91%. The results revealed that the fabricated MALAT1 biosensor was outstanding. Such it can be used for MALAT1 detection with receivable reproducibility.

Then, stability of the MALAT1 biosensor was analyzed. Storage time lasts 28 days and the temperature sets 4°C. Subsequently, those biosensors were measured each three days. The CV peak current of the MALT1 biosensor reduced slowly. Compare to the initial current, the final peak current maintained 98.14% after 28 days. It shows the acceptable stability of the MALAT1 biosensor.

**2.4 Detection of lncRNA MALAT1 in HCC cell lines**

Real application using the proposed biosensor was investigated by standard addition methods in HCC cell lines. HepG2 and PL-2 are classical and common liver cancer cell lines. So both cell lines were employed in the real application test. *rt*-PCR was used to provide a quantitative comparison. The results show that the relative expression of HepG2 and Hep3B are respectively 3.29±0.21 and 2.71±0.10 (Fig. S3). The results were supported by previous references [3,4].


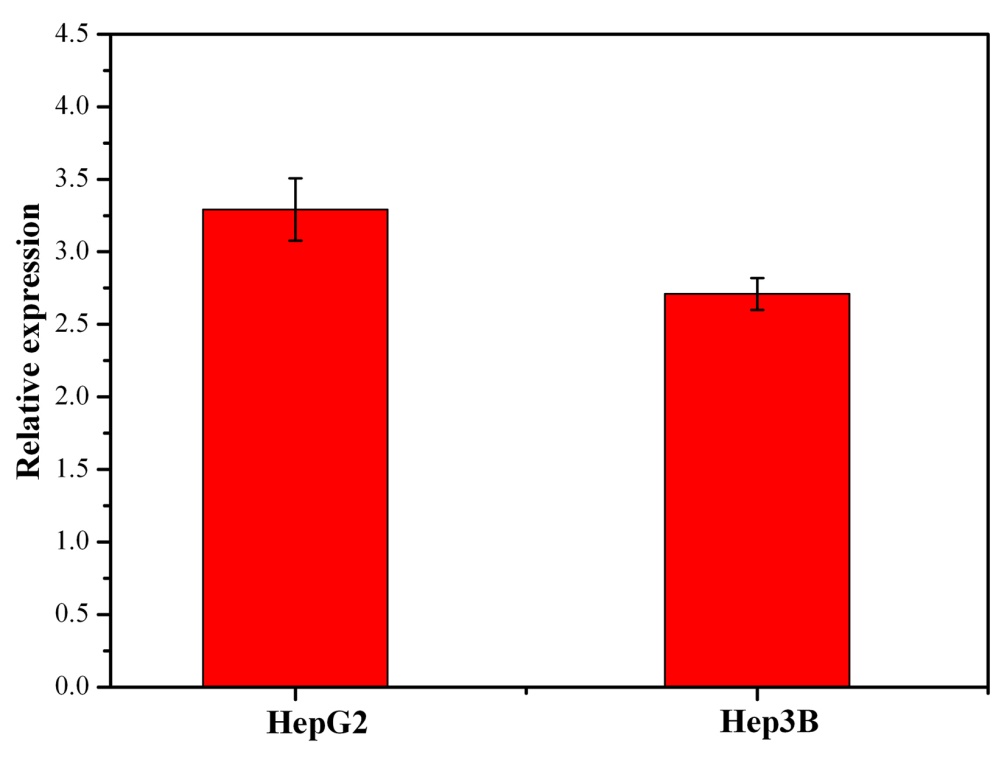


**Fig. S3** Expression of lncRNA MALAT1 in HCC cell lines (N=3).

Then, the MALATA1 expression of HepG2 and Hep3B were detected by proposed biosensor. The results revealed the absolute current of HepG2 is 15.17 μA and the Hep3B is 14.98 μA. Converting according to linear equation, the concentration of MALAT1 of HepG2 and Hep3B are respectively 807.49 and 591.06 pmol⋅mL^-1^. The changing trend of biosensor results are very similar to those detected by *rt*-PCR (Table S2), which indicated that the proposed biosensor was feasible for the determination of lncRNA MALAT1 and could satisfy the need for practical analyses.

**Table. S2** Determination of MALAT1 concentration of HCC cell lines

with proposed biosensors .

| HCC cell lines | *rt*-PCR  (relative expression) | Response  (μA) | Relative standard deviation (%) | Observed concentration  (pM/mL) |
| --- | --- | --- | --- | --- |
| HepG2 | 3.29 | -15.17 | 2.97 | 807.49 |
| Hep3B | 2.71 | -14.98 | 4.15 | 591.06 |

**References:**

1. Janyasupab M, Liu CW, Zhang Y, Wang KW, Liu CC (2013) Bimetallic Pt-M (M = Cu, Ni, Pd, and Rh) nanoporous for H2O2 based amperometric biosensors. Sensor Actuat B-Chem 179:209-214. doi:DOI 10.1016/j.snb.2012.09.099

2. Lokesh KS, Shivaraj Y, Dayananda BP, Chandra S (2009) Synthesis of phthalocyanine stabilized rhodium nanoparticles and their application in biosensing of cytochrome c. Bioelectrochemistry 75 (2):104-109. doi:DOI 10.1016/j.bioelechem.2009.02.005

3. Ji D-G, Guan L-Y, Luo X, Ma F, Yang B, Liu H-Y (2018) Inhibition of MALAT1 sensitizes liver cancer cells to 5-flurouracil by regulating apoptosis through IKKα/NF-κB pathway. Biochem Bioph Res Co 501 (1):33-40. doi:https://doi.org/10.1016/j.bbrc.2018.04.116

4. Li C, Miao RC, Liu SS, Wan Y, Zhang SM, Deng Y, Bi JB, Qu K, Zhang JY, Liu C (2017) Down-regulation of miR-146b-5p by long noncoding RNA MALAT1 in hepatocellular carcinoma promotes cancer growth and metastasis. Oncotarget 8 (17):28683-28695. doi:10.18632/oncotarget.15640
